# A modified technique of mega prosthesis revision on non-neoplastic patient: Case report

**DOI:** 10.1016/j.amsu.2020.08.036

**Published:** 2020-09-02

**Authors:** Yogi Prabowo, Didi Saputra Ramang, Syahdi Farqani, I Wayan Arya Mahendra Karda

**Affiliations:** a Oncology Consultant, Department of Orthopaedic and Traumatology, Cipto Mangunkusumo General Hospital, Faculty of Medicine-Universitas Indonesia, Indonesia; b Resident of Department of Orthopaedic and Traumatology, Cipto Mangunkusumo General Hospital, Faculty of Medicine-Universitas Indonesia, Indonesia

**Keywords:** Implant failure, Mega prosthesis, Endoprosthesis, Implant revisions, Case report

## Abstract

**Introduction:**

Mega prosthesis is mainly used for the treatment of the oncologic patient whose limb underwent salvage surgery that caused the limb to lose significant bone or soft tissue. In recent years, mega prosthesis can also be used to treat non-oncologic patients.

**Presentation of case:**

We presented a case of a 40-year-old male with chief complain of pain on the right knee 5 months before admission. Four years previously, the patient sustained motor vehicle accident that fractured his head of femur dan distal femur. He underwent 2-staged surgery for his femoral head and distal femur. However, he presented a year later with signs of non-union and finally underwent mega prosthesis surgery on his distal femur. During his follow up, he experienced a fracture on his prosthesis 3 years later and was referred to our institution. Physical examination shows deformity and slight varus on the right knee, and limited range of motion. The patient then underwent implant revisions.

**Discussion:**

After 12 months of post revision surgery follow-up, the patient was able to walk independently**.** Our patient has not had any sign or episode of failure after the follow up for 12 months. According to literature, the incidence of failure is mostly at 48–72 months post implantation.

**Conclusion:**

The problem for this patient maybe caused by the mechanical fatigue of the implant due to stress addressed to the implant. Our current technique of revisions procedure hopefully will enhance the power of the mega prosthesis for further usage.

## Highlights

• Mega prosthesis is mainly used for the treatment of the oncologic patient whose underwent salvage surgery causing the limb to lose significant bone or soft tissue.
• In recent years, mega prosthesis can also be used to treat non-oncologic patients.
• Mega prosthesis revision using a modified technique resulted in good functional outcome.

## 1 Introduction

Mega prosthesis or endoprosthesis, is a well-established modality for reconstruction treatment of tumors [[1], [2], [3], [4]]. The primary function of mega prosthesis is to provide functional joint of limbs for the patient whose limb has significant bone or soft tissue loss. Thus, the primary indication for the mega prosthesis is for the oncologic patient who must undergo limb salvage surgery that is expected to have major bone or soft tissue defect.

However, nowadays the indication for mega prosthesis have been expanded to the treatment of non-oncologic patients, which is also associated with severe bone loss and failed arthroplasty, comminuted fractures in the elderly with poor bone quality, and resistant non-union [1,5]. Mega prosthesis for non-oncologic patient is a viable option for the treatment for the compromised bone stock and heavily impaired structural integrity caused by multiple etiologies.

In the meta-analysis done by Henderson et al. [6], the rate of failure of the prosthetic component in distal femur reconstruction was 6.3% and 2–12% in proximal tibia. Overall, 4.8% of mega prosthesis broke and require revisions. The main indication for the revisions is failure of the mega prosthesis, which can be classified into 5 types: type 1 (soft tissue failure), type 2 (aseptic loosening), type 3 (structural failure), type 4 (infection), and type 5 (local tumor recurrence). We present a 40-year-old male with implant failure of mega prosthesis treated in our institution. Informed consent has been given by the patient to be reported in a case report.

This report is complaint with consensus-based surgical case report guidelines, SCARE Guidelines [7].

### 1.1 Presentation of Case

A 40-year-old male was presented to our institution with pain on right knee since 5 months prior to admission. The pain was present while the patient stands and worsened by walking. He had a history of motorcycle accident in August 2014. The patient did not seek any medication at first. But because of increasing pain, he was brought to the nearest local hospital. There was no known family history of the same condition. He had been told that the patient's femoral head was protruded to pelvic cavity and his distal femur was fractured. In August 2014 he had his first stage surgery of the hip. Later, on March 2015 he had his second stage surgery consisted of open reduction and internal fixation of the distal femur. Nine months after the surgery (November 2015), no signs of union were detected from the patient, and so he had his implant removed. In January 2016, he underwent distal femur surgery by mega prosthesis. After surgery, the patient can walk and do daily activities normally. No limitation was found 3 years after, however, on April 2019, he complained knee pain after a “crack” sound was heard while praying. The patient was then referred to our institution for further treatment.

**Fig. 1:**
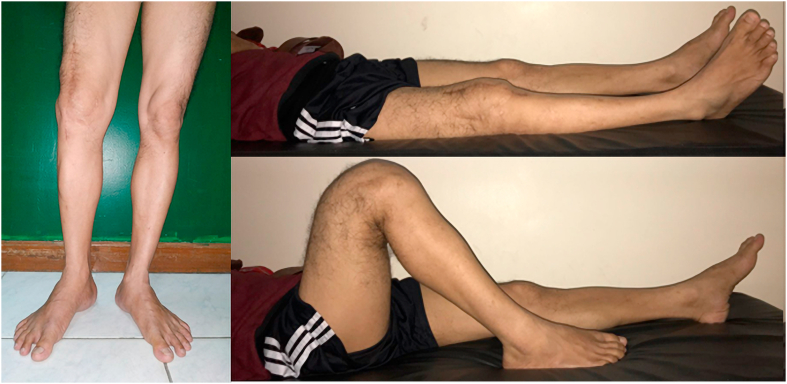
spl A spl Clinical condition of both knees.

**Fig. 2:**
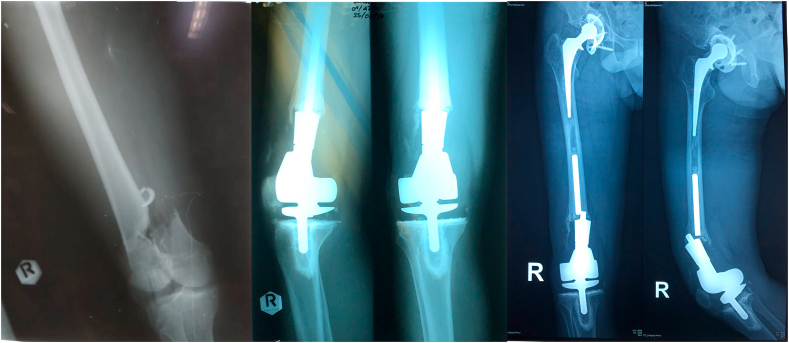
Serial X-ray of the right knee.

From the physical examination, his right knee was slightly deformed and varus, scar from previous operation was seen, no swelling nor muscle atrophy detected. The patient felt localized tenderness at the knee with visual analogue scale (VAS) of 3. His range of motion of right knee was limited with extension-flexion 0–110°. The clinical condition of the patient was presented in Fig. 1. The complete blood count was normal with Hemoglobin level of 15,4 g/dl, total leukocyte of 8,2 × 10^9^/L, and thrombocyte count of 330.000/μl. Abnormal values were found in ESR and CRP examinations with 55mm/hour and 13,3mg/L respectively. Serial X-ray of the right knee is displayed in Fig. 2. The patient was diagnosed implant failure of right knee post mega prosthesis and total hip replacement. He was then planned to perform implant revision surgery.

**Fig. 3:**
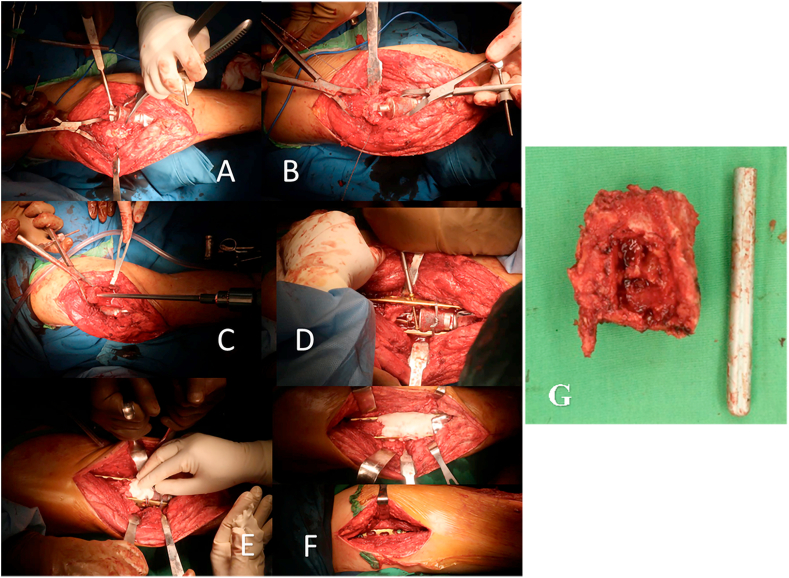
Intraoperation documentary. A. Implant exposure, B. Distal femoral osteotomy, C. Femoral reaming, D. Screw insertion, E. Cement insertion, F. Final construct, G. Osteotomized part of the distal femur.

**Fig. 4:**
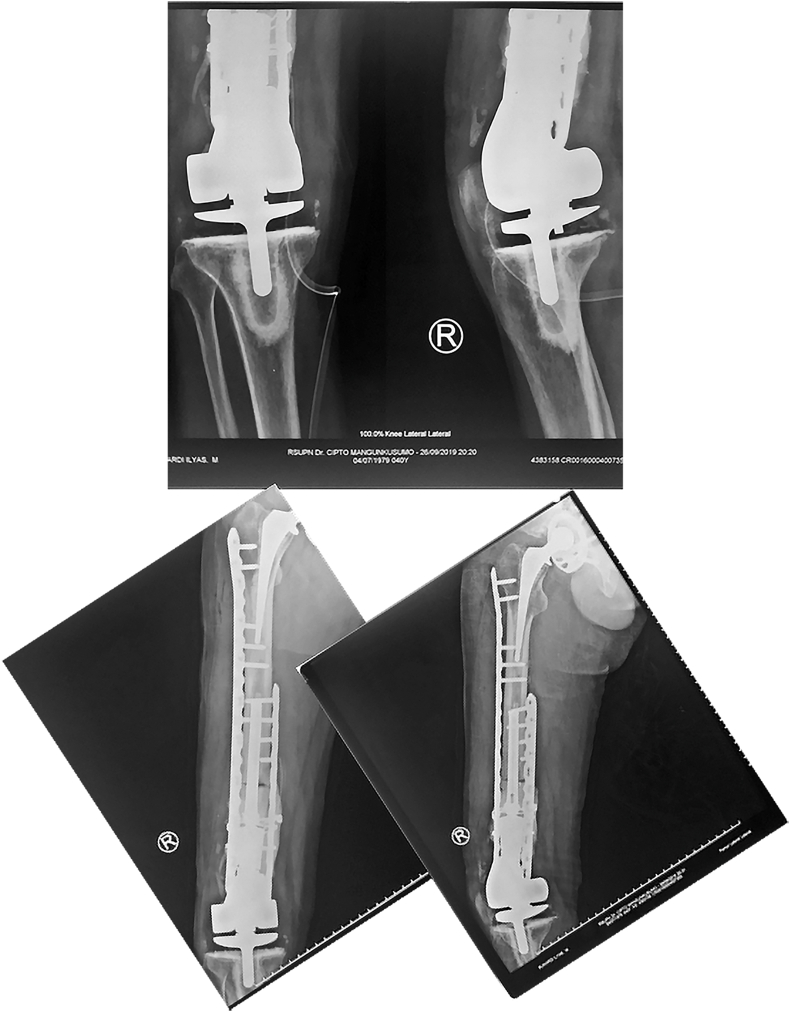
Post-operative x-ray in June 2020.

The procedure was performed by our senior Orthopaedic oncology surgeon. Before the surgery, cefoperazone sulbactam was given, as prophylactic antibiotics, an hour before the surgery began. At the operating theatre, the patient was in supine position and epidural anesthesia was administered. Aseptic and antiseptic procedure was performed before incision. Incision was made on top of previous surgery scar and incised until the implant was exposed. Intraoperatively, the implant was confirmed failure at the distal femoral stem (Fig. 3A). The wound was cleansed with NaCl 0.9% then osteotomy was performed at the distal femur (Fig. 3B). Osteotomy was performed because there was difficulty in removing the mega prosthesis. The osteotomized part, was later reconstructed with plate and screw fixation. The failed implant then extracted using extractor and the remaining femoral stem was removed and the proximal femur was reamed. After reaming, a reconstruction of the failed mega prosthesis was performed by using K-nail sized 12. After that, double broad plate was inserted at the medial and lateral side of the femur to fixate the distal and proximal femur. Later, the construction was enhanced by two cerclage wires at the intersection of distal femoral stem of the implant and double plate. The plate was then fixated by screws and the bone cement was applied (Fig. 3D). After the operation was complete, the wound then cleansed by NaCl 0,9% and sutured. Post operation x-ray was obtained (Fig. 4). There was no neurological injury, wound infections, and other complications after the surgery. The patient was then advised to use Robert Jones bandage until the soft tissue swelling subsided approximately 3–7 days and was encouraged to do non weight bearing mobilization using bilateral crutches.

After discharged, the patient was advised to do rehabilitation and routine control to policlinic. The patient increased his load bearing into partial weight bearing after 3 weeks. Post-operative compliance of the revision procedure from the patient was good. The patient followed the weight bearing course accordingly and routinely controlled to the polyclinic.

**Fig. 5:**
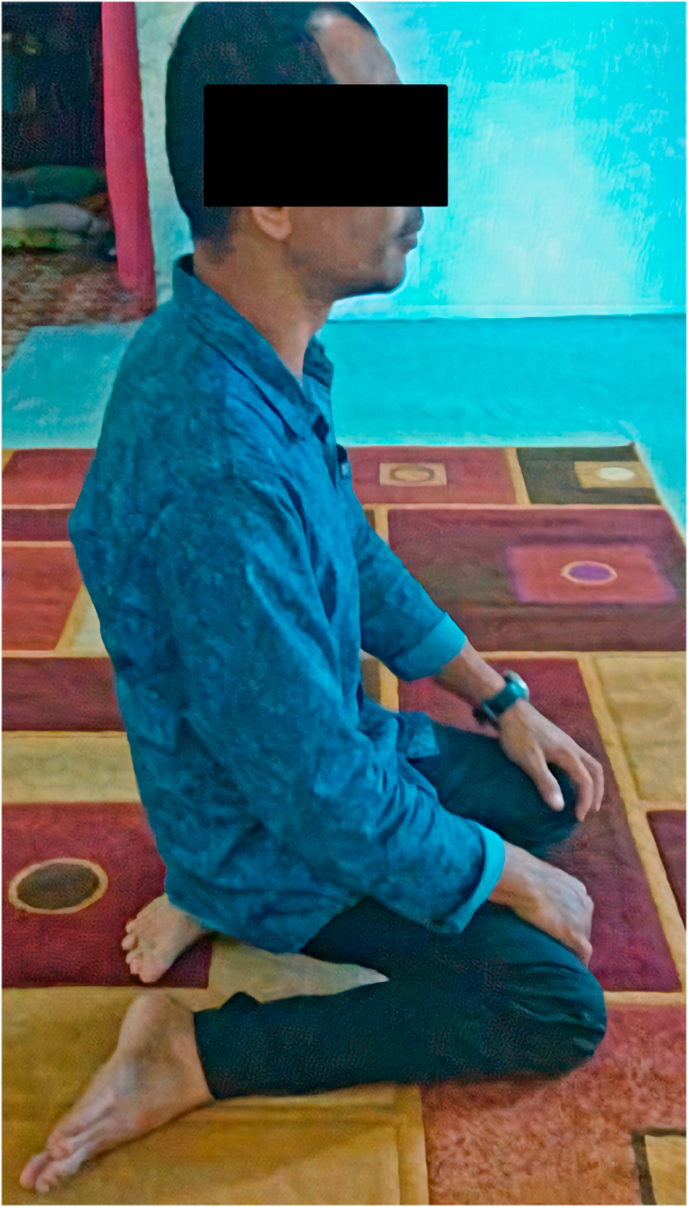
Knee flexion after a year post-operation follow-up.

After 12 months follow-up, the patient was able to walk independently without any supporting tools. His knee was able to flex up to 120° (Fig. 5). The patient felt better overall and no longer felt pain on his knee. Twelve months after the procedure, the patient exhibited satisfactory results from the procedure, where the pain resides, and the wound recovered appropriately with good functional outcome.

## 2 Discussion

Mega prosthesis for the treatment for non-oncologic cases is still viable option although the complication and survival rates of these prostheses are inferior compared to primary arthroplasty [1]. The usage of the mega prosthesis for the revision knee arthroplasty allows limb salvage with acceptable outcome and rather high complication rate such as infection and reduced joint motion, but still an exceptional indication [2,8,9]. In spite of that, the application of mega prosthesis is still viable option for oncologic patient who has to undergo limb salvage surgery. One of the main indications for the application of mega prosthesis in non-oncologic patient is to cover the bone loss. In our case, our patient suffers great bone loss due to previous revision of arthroplasty, therefore according to literature the patient was eligible for the application of mega prosthesis. Unlike other types of implant, mega prosthesis have higher incidence of complications and failures, making revision surgery relatively frequent [10]. Failure in mega prosthesis can be classified into five types of classification: soft tissue failure (Type 1), aseptic loosening (Type 2), structural fracture (Type 3), infection (Type 4), and local tumor recurrence (Type 5) [6,10].

Soft-tissue failure is functional deficiencies of the soft-tissue attachments to the implant that require re-operation due to disruption of periarticular ligamentous and tendinous restrains. This type of failures accounted for 12% of all failures, most found around the shoulder and hip. Type 2 failure or aseptic loosening account for 4.9–9.6% of incidence and depends on the reconstruction site, with the highest rates of loosening in distal femoral replacement [10]. Type 3 failure in distal femur reconstruction happens in 6.3% of the cases, whereas in the tibia is 2–12%. Infection or type 4 failure is heavily associated with prosthetic joint infection (PJI). The most common pathogen for this type of infection is *Staphylococcus aureus*, which account for approximately 50% of cases [6].

The long-term survival of the implant is also good. Toepfer et al. [11], published a retrospective review of the patient treated with total femoral replacement for non-oncologic conditions from 1995 to 2015, which stated that the outcome of the patient mainly depend on the age at reconstruction not on the indication. From his review, the overall failure rate was 72%, with the most common failure mechanism was Type 1 (soft tissue), followed by Type 4 (infection) and type 3 (mechanical failure) [10,11]. Nevertheless, the long-term outcome for the elderly for the mega prosthesis is still good with low rate of complications [10,12]. According to literature, the incidence of failure is mostly at 48–72 month post implantation [13]. Evans et al. also reported after three years of follow up no patient, from total of 10 patients, had prosthetic failure [14]. Longer survival was reported by Biau et al. where median of mega prosthesis survival rate was up to 130 months following femoral resection and 117 months following median resection [4]. The problem for our patient maybe caused by the mechanical fatigue of the implant due to stress addressed to the implant.

Poorer outcome may be caused by several factors such as: immunosuppression of patients with oncologic diseases, extensive resection of the bony and soft tissue around the knee, longer operative time, and general patient conditions [12]. Our patient has not had any episode of failure after the follow up for 12 months post revision surgery. A long-term follow-up needs to be done to evaluate the outcome.

Calori et al. had stated that to achieve good quality range of movement in mega prosthesis patient was a big problem. It was because scar adhesions related to past failures, joint stiffness due to torsional deformities and joint degeneration, and muscle retraction with relative depletion of contractile function and muscle mass were all present and need to be addressed [15]. In our patient, we were able to achieve good ROM on the right knee, as the patient were able to achieve maximum knee flexion. We believe that our operation technique was one of the factors contributing to return of function. The previous fractured mega prosthesis stem was removed by osteotomizing the distal femur. After extraction, the osteotomized part was put back into the construct by inserting K nail, and plate and screw. The revision stem stability was enhanced by K nail, plate, and screw system until the proximal femur. Finally, bone cement was applied to stabilize the implant. The incidence of mega prosthesis failure is mainly due to the age at the surgery of mega prosthesis. In literature there is no clear support regarding method of stem fixation, but cementless fixation seems to improve the bone in-growth, which seems to lead to low aseptic loosening rate.

## 3 Conclusion

Despite the advances in materials and implant designs, a system of mega prosthesis still has a higher rate of complications such as failures. The problem for this patient maybe caused by the mechanical fatigue of the implant due to stress addressed to the implant. Our current technique of revisions procedure hopefully will enhance the power of the mega prosthesis for further usage.

## Funding sources

The authors report no external source of funding during the writing of this article.

## Ethical approval

Written informed consent was obtained from the patient for publication of this case report and accompanying images. A copy of the written consent is available for review by the Editor-in-Chief of this journal on request.

### Provenance and peer review

Not commissioned, externally peer reviewed.

## Declaration of competing interest

None.
